# *CLEC4E* as a molecular biomarker in systemic lupus erythematosus: integrating bioinformatics and clinical data to assess its prognostic value

**DOI:** 10.3389/fimmu.2025.1617878

**Published:** 2025-09-30

**Authors:** Xiaoxia Ma, Huan Zhang, Jiana Li

**Affiliations:** ^1^ Department of Laboratory Medicine, Tianjin Academy of Traditional Chinese Medicine Affiliated Hospital, Tianjin, China; ^2^ Department of Gastroenterology, No. 983 Hospital of People's Liberation Army Joint Logistics Support Force, Tianjin, China

**Keywords:** SLE, CLEC4E gene, biomarker, disease activity, diagnosis and prognosis

## Abstract

**Objective:**

This study explores the prognostic value of the *CLEC4E* gene in systemic lupus erythematosus (SLE) through bioinformatics analysis and evaluates its role in disease diagnosis and progression.

**Methods:**

Gene expression datasets related to SLE (GSE17755, GSE50772, and GSE61635) were obtained from the GEO (Gene Expression Omnibus) database. Intersection analysis was performed using the Jvenn tool with a screening threshold of |log2FC| > 1 and P< 0.05 to identify differentially expressed genes (DEGs). The resulting DEGs were then cross-referenced with immune-related genes in the GeneCards database (relevance score > 8) to further prioritize candidates with immunological relevance. Peripheral blood from 360 SLE patients and 360 healthy controls was collected for *CLEC4E* expression analysis via RT-qPCR. Disease activity was evaluated using the SLEDAI score, and patients were grouped accordingly. Pearson and Spearman correlation analysis to investigate the relationship between *CLEC4E* and immune indicators. Logistic regression and *ROC* analyses were conducted to assess diagnostic and prognostic value. Kaplan-Meier analysis evaluated survival outcomes.

**Results:**

Bioinformatics analysis identified six SLE-related DEGs, namely *ISG15*, *HERC5*, *TNFAIP6*, *IFIT3*, *OASL*, and *CLEC4E.* Further intersection with immune-related genes from the GeneCards database (relevance score > 8) ultimately highlighted *CLEC4E* as the key gene for clinical validation. The expression level of *CLEC4E* was significantly higher in SLE patients compared with healthy controls. *ROC* analysis showed good diagnostic performance (AUC = 0.7744). *CLEC4E* expression was higher in active SLE, and multivariate analysis identified *CLEC4E*, C3, C4, ANA, and anti-dsDNA as independent predictors of disease activity. *CLEC4E* demonstrated moderate diagnostic value for distinguishing active from inactive disease (AUC = 0.6360). Higher *CLEC4E* expression was associated with worse prognosis (*P* = 0.0002). The combined diagnostic performance with other biomarkers (C3, C4, ANA, anti-dsDNA) showed a remarkable AUC of 0.9407.

**Conclusion:**

*CLEC4E* is a potential biomarker for SLE diagnosis, disease activity assessment, and prognosis evaluation.

## 1 Introduction

Systemic lupus erythematosus (SLE) is a chronic autoimmune disease characterized by multisystem involvement and diverse clinical manifestations. It exhibits a relatively high global incidence, particularly among female patients (1). The pathogenesis of SLE is complex, involving interactions among genetic, environmental, hormonal, and immune factors (2). Despite significant progress in understanding the mechanisms of SLE in recent years, particularly through genome-wide association studies (GWAS) that have identified multiple susceptibility genes such as *IRF5, STAT4*, and *TNFSF4*, the disease’s complexity and heterogeneity continue to pose significant challenges for early diagnosis, effective treatment, and prognosis management (3, 4). Therefore, there is an urgent need to identify novel pathogenic genes and reliable molecular biomarkers to improve our understanding of disease mechanisms and clinical outcomes.

C-type lectin domain family 4 member E (*CLEC4E*), also known as MINCLE, is a pattern recognition receptor (PRR) that plays a pivotal role in host immune responses. It regulates inflammatory reactions by recognizing pathogen-associated molecular patterns (PAMPs) and damage-associated molecular patterns (DAMPs) (5, 6). *CLEC4E* is primarily expressed in macrophages and dendritic cells. In these cells, it mediates the release of inflammatory cytokines by interacting with downstream signaling pathways, such as the Syk-CARD9 pathway. This activation contributes to infection control, tissue damage, and the pathogenesis of autoimmune diseases (7, 8). Although recent studies have highlighted *CLEC4E’s* involvement in inflammation regulation, its specific role in SLE remains largely unexplored, particularly in terms of its contributions to immune dysregulation and disease progression. In addition to its role in SLE, *CLEC4E* has been implicated in other autoimmune diseases, such as rheumatoid arthritis, Sjögren’s syndrome, and multiple sclerosis (9–11). Studies have shown that *CLEC4E* influences disease mechanisms in these conditions by modulating immune responses and promoting inflammation (12). However, its precise role in SLE and its connection to disease advancement and immune dysregulation remain largely uninvestigated.

Abnormalities in the innate immune system are considered crucial in SLE pathogenesis. In particular, dysregulated PRR-mediated signaling pathways, which may drive autoimmune activation and tissue damage (13, 14). As an emerging PRR, *CLEC4E*’s potential as a functional target warrants further investigation.

In summary, this study aims to assess *CLEC4E* expression patterns in SLE and their associations with clinical indicators through bioinformatics analysis and clinical datasets. The study hypothesizes that abnormal *CLEC4E* expression is associated with the pathogenesis of SLE, particularly immune dysregulation and tissue damage. Furthermore, through multivariate analyses and ROC curve evaluations, the diagnostic and prognostic value of *CLEC4E* as a potential biomarker for SLE will be validated, providing a reference for early diagnosis and targeted therapy for SLE.

## 2 Materials and methods

### 2.1 Bioinformatics analysis

Gene expression data related to systemic lupus erythematosus (SLE) were retrieved from the Gene Expression Omnibus (GEO) database (GSE17755, GSE50772, GSE61635). Jvenn (http://jvenn.toulouse.inra.fr/) was used for intersection analysis of these three SLE-related datasets. The screening criteria for differentially expressed genes (DEGs) were set as |log2FC| > 1 and *P*< 0.05. DEGs obtained from each dataset were intersected to identify robust SLE-associated genes. Subsequently, the identified DEGs were cross-referenced with immune-related core genes retrieved from the GeneCards database using the keyword “Immunity” (relevance score > 8), in order to further prioritize candidate genes with immunological significance for downstream validation.

### 2.2 General data

A cohort of 360 SLE patients treated at our hospital between March 2023 and January 2025 was included in the study. The group consisted of 312 women and 48 men, with a mean age of 38.23 ± 5.60 years. The inclusion criteria were as follows: (1) meeting the 2019 classification criteria for SLE established jointly by the European League Against Rheumatism (EULAR) and the American College of Rheumatology (ACR) (15); (2) age >18 years; and (3) complete clinical data. The exclusion criteria were as follows: (1) coexisting other types of autoimmune diseases (e.g., rheumatoid arthritis); (2) coexisting infectious diseases; and (3) malignancies or pregnancy.

A control group was established consisting of 360 healthy individuals undergoing routine physical examinations during the same period. This group included 298 females and 62 males, with an average age of 38.42 ± 5.59 years. The healthy participants met none of the diagnostic criteria for SLE, had no history of major diseases, and had no personal or family history of autoimmune diseases.

### 2.3 Assessment of disease activity

The severity of disease was assessed using the Systemic Lupus Erythematosus Disease Activity Index (SLEDAI) (16). Patients with SLE were classified into the active group (SLEDAI ≥ 5) and the inactive group (SLEDAI*<* 5).

### 2.4 Detection methods

Fasting venous blood samples (5 mL) were collected from each subject and centrifuged at 3000 revolutions per minute (r/min) for 10 minutes using a centrifuge with a rotor diameter of 1000 mm. Following centrifugation, 150 μL of serum was separated and used for immunological analyses. Serum levels of immunoglobulin A (IgA), immunoglobulin M (IgM), immunoglobulin G (IgG), complement components C3 and C4, and C-reactive protein (CRP) were quantified using immunoturbidimetric assays. Peripheral blood cell parameters, including white blood cell (WBC) count, hemoglobin (Hb), and platelet (PLT) count, were analyzed using a Sysmex fully automated modular hematology and body fluid analyzer. Erythrocyte sedimentation rate (ESR) was measured by infrared photo-optical colorimetry. ANA were detected by indirect immunofluorescence assay (IIFA), and anti-double-stranded DNA (anti-dsDNA) antibodies were determined using a line immunoassay (LIA). The 24-hour urinary protein excretion was assessed using a TBA-FX8 fully automated biochemical analyzer.

### 2.5 RT-qPCR detection of *CLEC4E* gene expression

Blood samples from all participants were collected in EDTA-containing tubes for anticoagulation. Peripheral blood mononuclear cells (PBMCs) were separated through Ficoll-Paque density gradient centrifugation (GE Healthcare, Chicago, USA). Total RNA was isolated from PBMCs with TRIzol reagent (Invitrogen, Carlsbad, CA, USA) following the manufacturer’s protocol. The RNA concentration and purity were determined using a Nanodrop 2000 spectrophotometer (Thermo Fisher Scientific, Waltham, MA, USA). The RNA was then reverse-transcribed into cDNA using a reverse transcription kit (Thermo Fisher Scientific). RT-qPCR was performed on a QuantStudio M7 Flex RT-qPCR system (Thermo Fisher Scientific) using the SYBR Green PCR Master Mix kit (Applied Biosystems, Foster City, CA, USA). The primer sequences for the RT-qPCR reactions were as follows: *CLEC4E* forward primer: 5′-CCTGTTTCATCACCAGATGTGT-3′, *CLEC4E* reverse primer: 5′-AACGCCCAGGAAATGGTGT-3′; *GAPDH* forward primer: 5′-TGCAACCGGGAAGGAAATGA-3′, *GAPDH* reverse primer: 5′-TTCCCGTTCTCAGCCTTGAC-3′. The PCR amplification was carried out with an initial denaturation at 95°C for 5 minutes, followed by 40 cycles consisting of denaturation at 95°C for 15 seconds, annealing at 60°C for 30 seconds, and extension at 72°C for 30 seconds. The relative expression levels of *CLEC4E* were calculated using the 2*^−^* ^ΔΔ^ *^Ct^* method and normalized to *GAPDH* as an internal control.

### 2.6 Evaluation of diagnostic value

ROC curve analysis was used to evaluate the diagnostic performance of *CLEC4E* expression levels in distinguishing between SLE patients and healthy controls, as well as between active and inactive SLE patients.

### 2.7 Prognostic analysis

SLE patients were categorized into low and high *CLEC4E* expression groups based on the average expression value. Prognosis was assessed over a 6-month follow-up period post-discharge. Evaluations included updated SLEDAI scores, which were interpreted as follows: Favorable prognosis: SLEDAI score ≤ 4. Unfavorable prognosis: Disease relapse or death. Disease relapse was defined as an increase in the SLEDAI score of more than 3 points compared to the score at the end of treatment. Disease relapse was verified by clinical physicians. Death cases only included those directly related to SLE, such as those caused by disease complications or organ failure. The relationship between *CLEC4E* expression levels and SLE patient prognosis was assessed using survival analysis with Kaplan-Meier curve analysis.

### 2.8 Statistical analysis

Statistical analyses were conducted using SPSS software version 26.0 (IBM Corp., Armonk, NY, USA) and GraphPad Prism version 8 (GraphPad Software, San Diego, CA, USA), and R software version 4.4.3 (R Foundation for Statistical Computing, Vienna, Austria) was used for interaction effect analysis. For continuous variables with normal distribution, differences between groups were compared using the independent-samples *t-*test. For continuous variables not following a normal distribution, the Mann-Whitney *U* test (rank-sum test) was used. Differences in categorical variables were analyzed using the chi-square test. Prognostic rates were analyzed using Kaplan-Meier survival curves. A value of *P*< 0.05 was considered statistically significant.

## 3 Results

### 3.1 Bioinformatics analysis reveals SLE-associated DEGs

**Figure 1:**
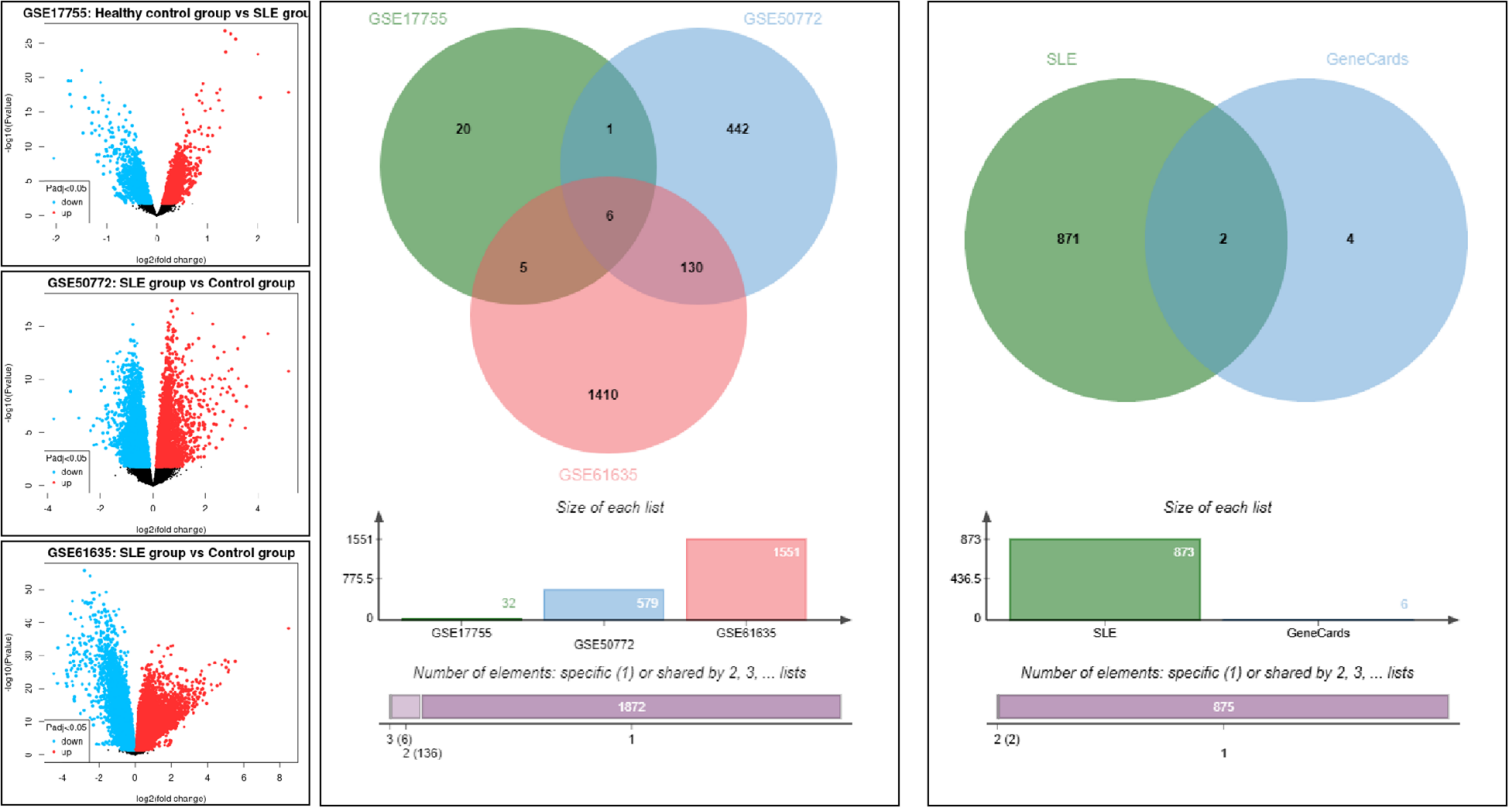
Bioinformatics analysis related to SLE.

Gene expression datasets related to SLE, including GSE17755, GSE50772, and GSE61635, were retrieved from the GEO database. Intersection analysis was conducted with the Jvenn tool, applying a screening criterion of |log2FC| > 1 and *P<* 0.05. A total of six DEGs were identified: *ISG15, HERC5, TNFAIP6, IFIT3, OASL*, and *CLEC4E*. To further prioritize genes with immunological relevance, the identified DEGs were cross-referenced with immune-related genes from the GeneCards database (relevance score > 8). Only *ISG15* and *CLEC4E* met these criteria. Considering that *ISG15* has already been extensively investigated in clinical studies of SLE and its role and clinical significance have been systematically elucidated, additional validation would provide limited novelty. In contrast, *CLEC4E* has been rarely reported in SLE, remains to be clinically validated, and is feasible for detection in peripheral blood, thus offering both innovation and translational potential. On this basis, *CLEC4E* was selected as the key gene for subsequent clinical validation. See **Figure 1**.

### 3.2 Comparison of general demographics

A comparison was made between the SLE patient group and the healthy control group (Control) regarding general demographic characteristics and lifestyle habits. No significant differences were observed between the two groups regarding age, gender ratio, BMI, smoking history, alcohol consumption, or education level (*P* > 0.05), suggesting comparability, as shown in **Table 1**.

**Table 1:** Comparison of general demographics [ $\bar{x}$ *± s, n (%)*].

Factor		Control Group	SLE Group	*T/x* ^2^	*P*
		(n=360)	(n=360)		
Age (years)		38.23 ± 5.60	38.42 ± 5.59	0.47	0.64
Gender	Male	62 (17.22%)	48 (13.33%)	2.10	0.15
	Female	298 (82.78%)	312 (86.67%)		
BMI (kg/m^2^)		21.63 ± 1.19	21.78 ± 1.31	1.58	0.12
Smoking History (Yes)		65 (18.06%)	52 (14.44%)	1.73	0.19
Drinking History (Yes)		70 (19.44%)	55 (15.36%)	2.08	0.15
Education Level	College or abov e	262 (72.78%)	249 (69.17%)	1.14	0.29
	High school or below	98 (27.22%)	111 (30.83%)		

### 3.3 Expression of *CLEC4E* in SLE

**Figure 2:**
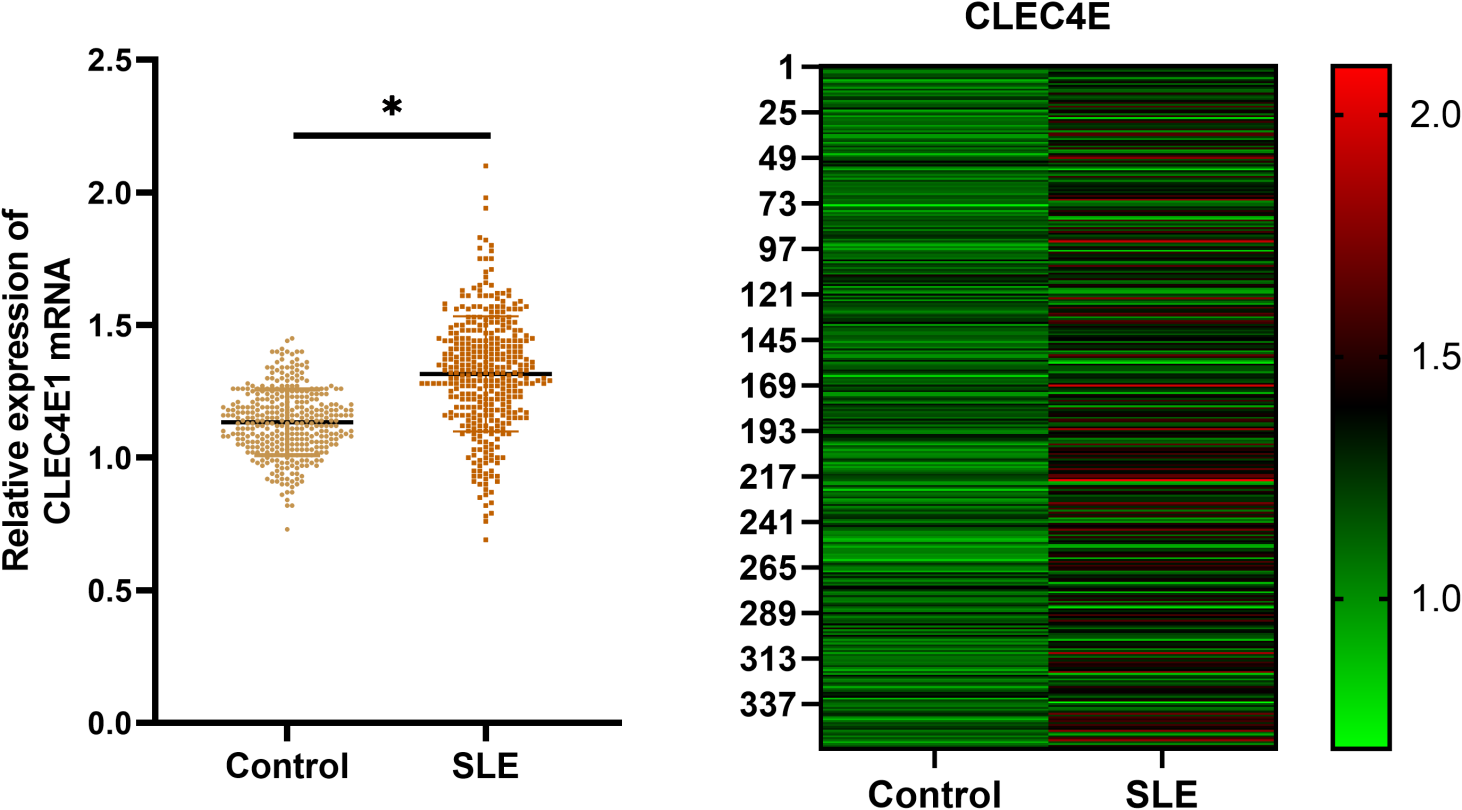
Expression of *CLEC4E* in healthy controls and SLE patients. The symbol * indicates a statistically significant difference between the two groups (*P < 0.05).

The expression of the *CLEC4E* gene was detected in both the SLE patient group and the healthy control group using RT-qPCR. The results indicated that *CLEC4E* expression was significantly elevated in the SLE group relative to the control group (*P<* 0.05). See **Table 2** and **Figure 2**.

**Table 2:** Comparison of *CLEC4E* expression levels [ $\bar{x}$ *± s*].

Factor	Control	SLE	*T*	*P*
	(n=360)	(n=360)		
*CLEC4E*	1.13 ± 0.12	1.32 ± 0.22	13.86	<0.0001

### 3.4 Diagnostic value of *CLEC4E*

**Figure 3:**
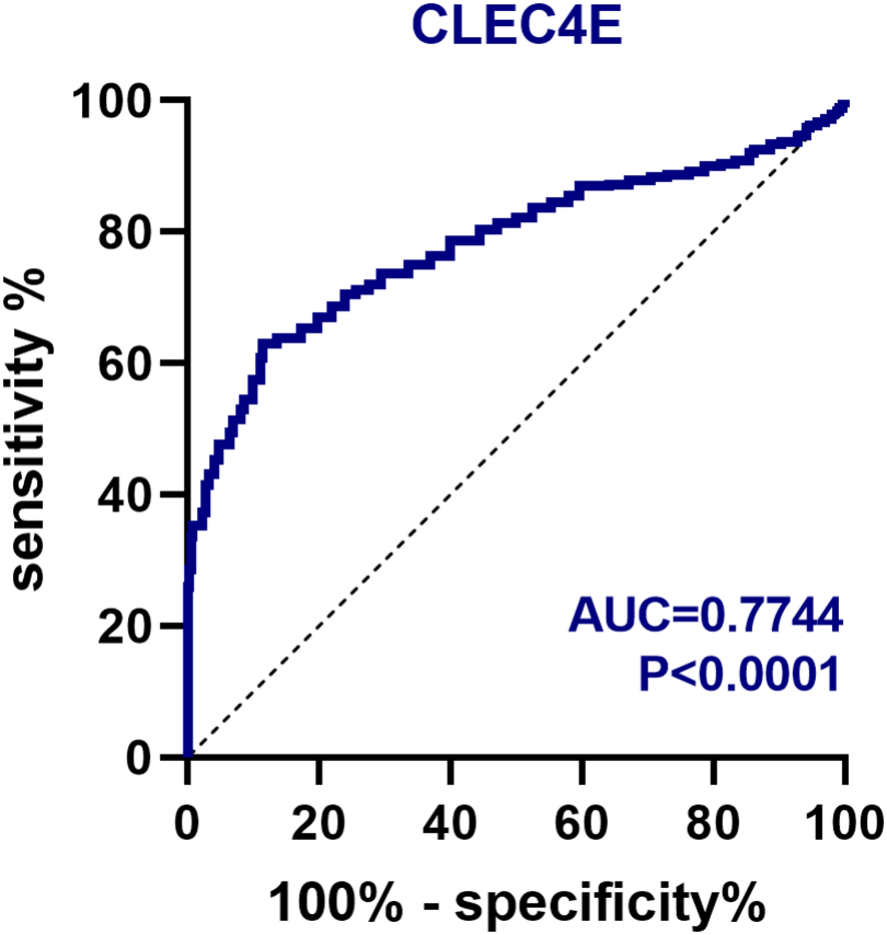
ROC curve analysis of the diagnostic value of *CLEC4E*.

The diagnostic value of *CLEC4E* in SLE patients was assessed using ROC curve analysis. The AUC for *CLEC4E* was 0.7744, with a sensitivity of 63.06% and a specificity of 86.39%, indicating that *CLEC4E* has high diagnostic performance for SLE. See **Table 3** and **Figure 3**.

**Table 3:** ROC curve analysis of *CLEC4E*'s diagnostic value.

Factors	AUC	Sensitivity (%)	Specificity (%)	Optimal cutoff value	95% CI
*CLEC4E*	0.7744	63.06%	86.39%	0.4945	0.7392-0.8096

### 3.5 Comparison of clinical characteristics between SLE patients with different disease activity

Based on the SLEDAI scores, SLE patients were divided into active and inactive groups. Clinical parameters were compared between the two groups. No significant differences were found in disease duration, WBC, HB, PLT, IgA, IgG, and IgM between the two groups (*P* > 0.05). However, the active group exhibited significantly elevated levels of immunological and inflammatory markers, ANA positivity, anti-dsDNA antibody levels, 24-hour urinary protein levels, ESR, and CRP. In contrast, serum levels of C3 and C4 were significantly reduced in this group (*P<* 0.05), indicating higher disease activity in active SLE patients. See **Table 4**.

**Table 4:** Comparison of clinical characteristics between SLE patients with different disease activity [M (Q_Min_, Q_Max_), $\bar{x}$ *± s, n (%)*].

Factors	Inactive group	Active group	*T*/*Z*/*x* ^2^	*P*
	(n=218)	(n=142)		
SLEDAI score	3 (0, 4)	9 (5, 12)	16.26	<0.0001
Disease Duration (months)	7.55 ± 1.84	7.74 ± 1.84	0.96	0.34
WBC (×10^9^/L)	6.90 ± 1.06	7.06 ± 1.26	1.32	0.19
HB (g/L)	124.72 ± 11.40	122.87 ± 12.70	1.44	0.15
PLT (×10^9^/L)	141.42 ± 18.34	139.10 ± 19.30	1.15	0.25
24h Urine Protein (g)	1.21 ± 0.14	1.26 ± 0.20	2.53	0.01
ESR (mm/h)	18.71 ± 3.62	19.73 ± 4.53	2.38	0.02
CRP (mg/L)	7.56 ± 1.22	7.95 ± 1.35	2.88	0.004
IgA (g/L)	2.45 ± 0.49	2.51 ± 0.53	1.17	0.24
IgG (g/L)	13.26 ± 1.17	13.50 ± 1.44	1.72	0.09
IgM (g/L)	1.13 ± 0.22	1.16 ± 0.30	0.86	0.39
C3 (g/L)	0.99 ± 0.14	0.77 ± 0.19	12.54	<0.0001
C4 (g/L)	0.17 ± 0.05	0.11 ± 0.03	10.83	<0.0001
ANA Positive [n (%)]	132 (61.11%)	112 (78.87%)	12.45	<0.0001
Anti-dsDNA Antibody (IU/ml)	18.82 ± 4.51	24.18 ± 5.34	10.24	<0.0001

### 3.6 Correlation analysis between *CLEC4E* and immune-related indexes in SLE patients

**Figure 4:**
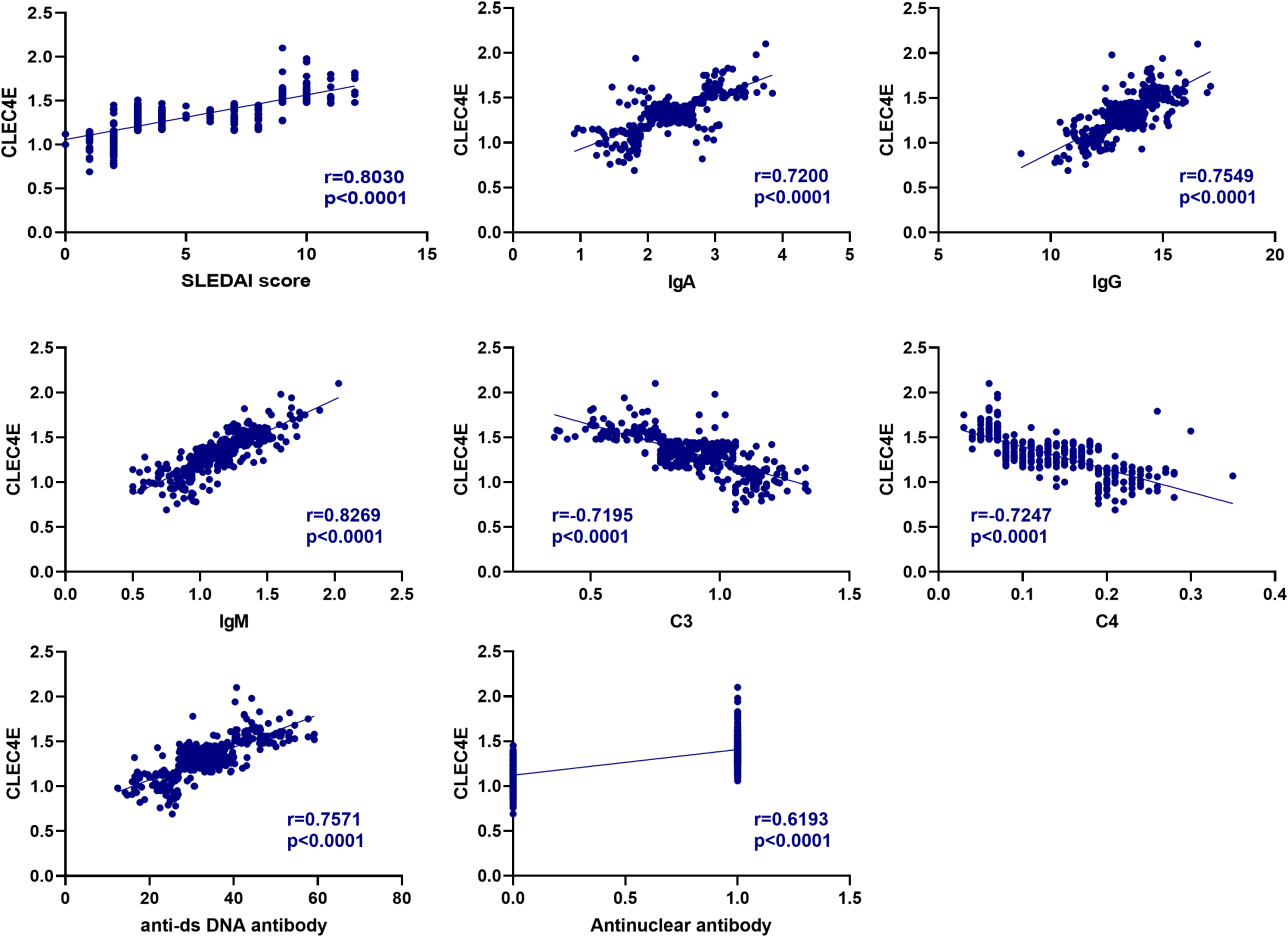
Pearson and Spearman analyses for correlation between *CLEC4E* and immune-related indexes.

The correlation between *CLEC4E* and SLEDAI score, IgA, IgG, and IgM was 0.7200, 0.7549, and 0.8269, respectively (*P<* 0.0001), and negatively correlated with C3 and C4, with correlations of -0.7195 and -0.7247, respectively (*P<* 0.0001). In addition, the correlation of *CLEC4E* with ANA positivity and anti-dsDNA antibody was 0.7571 and 0.6193 (*P<* 0.0001), respectively (**Table 5** and **Figure 4**).

**Table 5:** Correlation analysis between *CLEC4E* and immune-related indexes in SLE patients.

Factors	*CLEC4E*	
	*r*	*p*
SLEDAI score	0.8030	<0.0001
IgA	0.7200	<0.0001
IgG	0.7549	<0.0001
IgM	0.8269	<0.0001
C3	-0.7195	<0.0001
C4	-0.7247	<0.0001
ANA Positive (%)	0.7571	<0.0001
Anti-dsDNA Antibody	0.6193	<0.0001

### 3.7 Expression difference of *CLEC4E* in SLE patients with different disease activity

**Figure 5:**
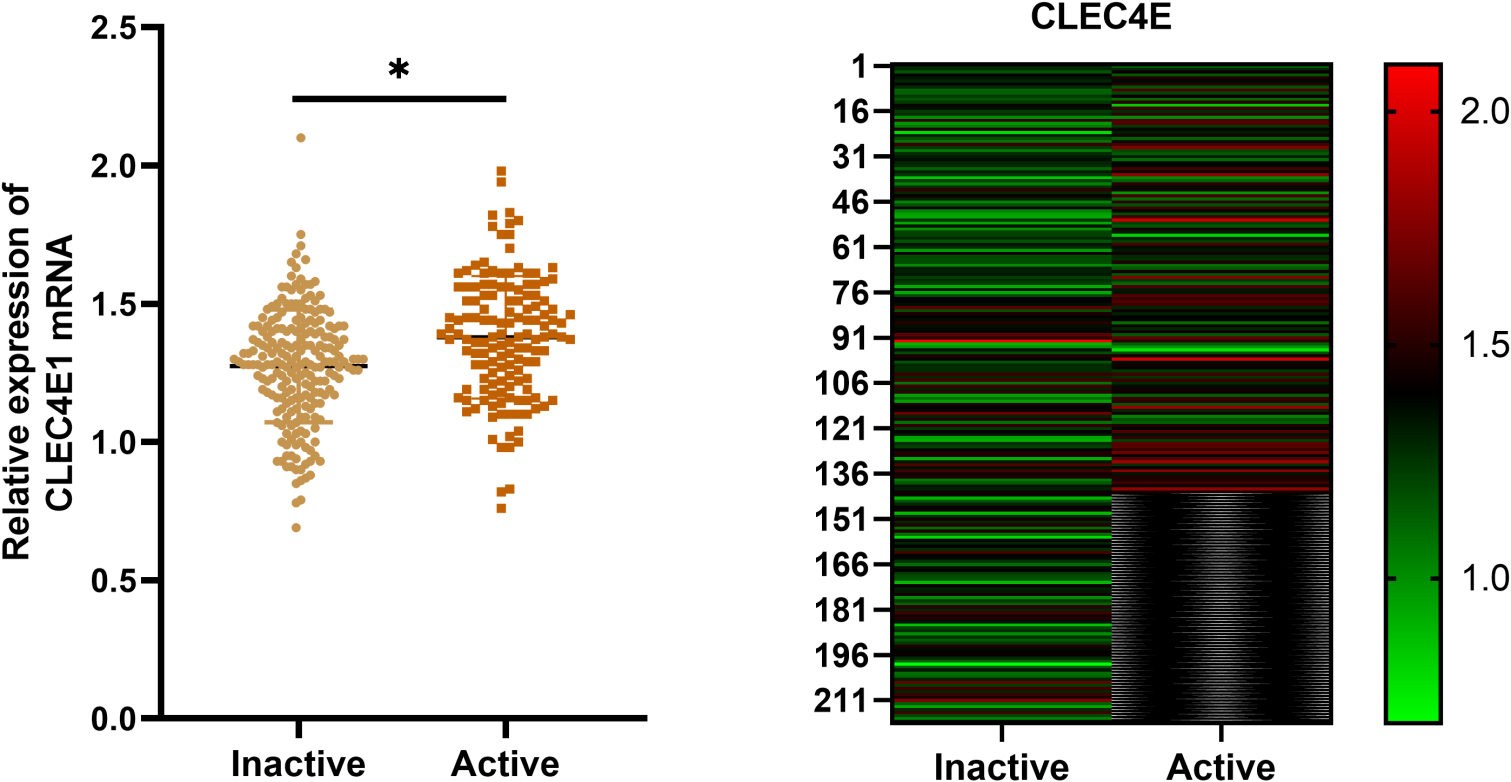
Expression of *CLEC4E* in SLE patients with different disease activity. The symbol * indicates a statistically significant difference between the two groups (*P < 0.05).

*CLEC4E* expression was significantly higher in the active SLE group compared to that in the inactive group (*P*< 0.05). This finding suggests that *CLEC4E* may be closely related to the disease activity of SLE. See **Table 6** and **Figure 5**.

**Table 6:** Expression of *CLEC4E* in SLE patients with different disease activity [ $\bar{x}$ *± s*].

Factors	Inactive group	Active group	*T*	*P*
	(n=218)	(n=142)		
*CLEC4E*	1.27 ± 0.21	1.39 ± 0.21	5.73	<0.0001

### 3.8 Multivariate analysis of independent risk factors for disease activity in SLE patients

A multivariate logistic regression analysis was conducted to explore potential independent risk factors for disease activity in SLE. The factors with a *P*-value of<0.05 from the previous differential analysis were subjected to collinearity diagnostics. The results showed that the variance inflation factor (VIF) for all independent variables was less than 10, and the tolerance values for each variable were greater than 0.1, indicating that there was no significant linear correlation between the independent variables. Based on the conventional criteria for VIF and tolerance, no potential risk for multicollinearity was observed, as shown in **Table 7**. The regression analysis revealed that C3, positive ANA, anti-dsDNA antibodies, and *CLEC4E* expression levels were independent risk factors for SLE disease activity (*P<* 0.05). This suggests the potential prognostic value of *CLEC4E* in SLE progression. See **Table 8**.

**Table 7:** Collinearity diagnosis.

Variable	Tolerance	VIF
Constant	–	–
Urine protein	0.978	1.023
ESR	0.96	1.041
CRP	0.979	1.021
C3	0.862	1.16
C4	0.9	1.111
ANA positivity	0.981	1.02
Anti-dsDNA antibody	0.892	1.121
*CLEC4E*	0.933	1.071

**Table 8:** Multivariate analysis of risk factors for disease activity in SLE patients.

Variable	*β*	SE	Wald *x* ^2^	*P-value*	Exp (B)
Constant	0.762	2.702	0.080	0.778	0.467
Urine protein	1.830	1.169	2.452	0.117	6.235
ESR	0.086	0.047	3.325	0.068	1.090
CRP	0.139	0.140	0.980	0.322	1.149
C3	9.165	1.400	42.833	0.000	0.000
C4	29.740	4.774	38.814	0.000	0.000
ANA positivity	0.948	0.403	5.523	0.019	0.388
Anti-dsDNA antibody	0.232	0.041	32.807	0.000	1.261
*CLEC4E*	2.865	0.858	11.137	0.001	17.544

**Figure 6:**
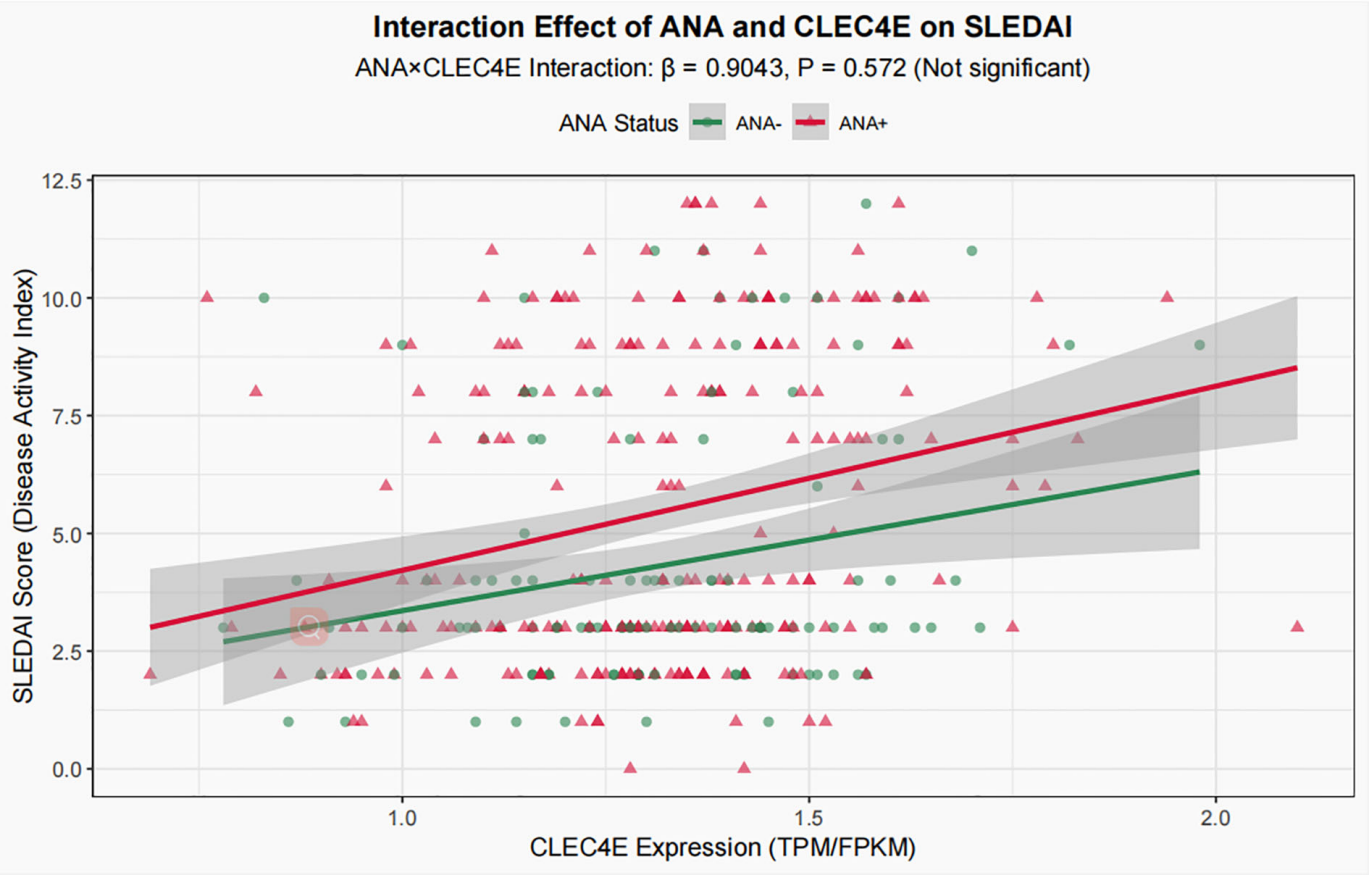
Interaction analysis of ANA and *CLEC4E*.

To explore the potential interaction between *CLEC4E* expression and ANA status, an interaction term (ANA × *CLEC4E*) was added to the aforementioned multivariate model. The analysis showed that the regression coefficient of the interaction term was 0.9043 with a P value of 0.572, indicating that the effect of *CLEC4E* on disease activity (SLEDAI) did not differ significantly between ANA-positive and ANA-negative patients (**Figure 6**). Therefore, the interaction term was not included in the main-effect model, and subsequent analyses and conclusions were based on the multivariate model without the interaction term.

### 3.9 Diagnostic value of *CLEC4E* in SLE at different disease stages

**Figure 7:**
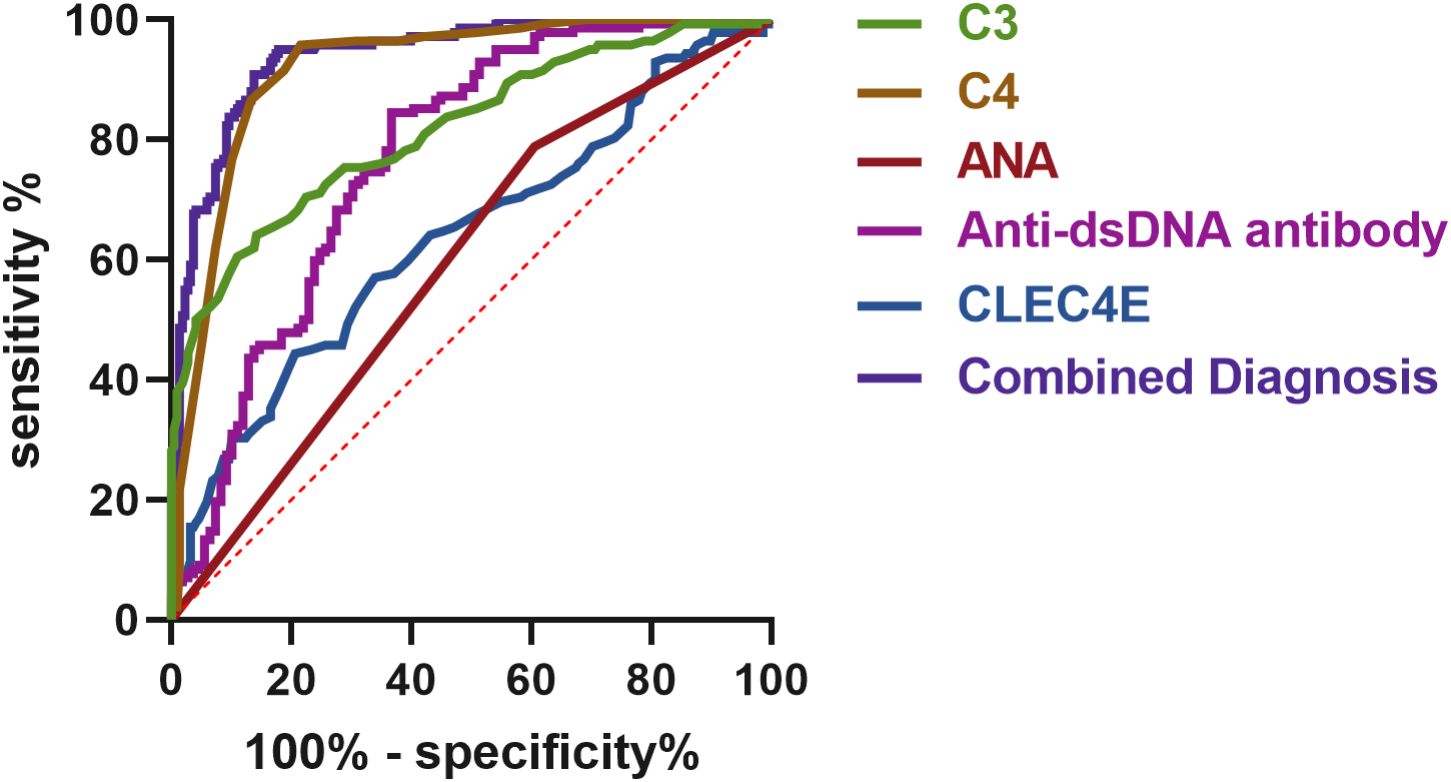
ROC curve analysis of the diagnostic value of *CLEC4E* in SLE at different disease stages.

The diagnostic value of *CLEC4E* in different disease activity phases of SLE was evaluated using ROC curve analysis. The results showed that *CLEC4E* had an AUC of 0.6360, a sensitivity of 44.37%, and a specificity of 79.36%, indicating its potential diagnostic value in assessing SLE activity, particularly in distinguishing patients with high specificity. Although *CLEC4E*’s standalone diagnostic performance has certain limitations, its diagnostic capacity significantly improves when combined with other markers (such as C3, C4, ANA, and anti-dsDNA antibodies). The combined diagnostic performance showed a remarkable AUC of 0.9407, with a sensitivity of 95.07% and a specificity of 82.11%, further highlighting *CLEC4E*’s potential in SLE diagnosis, especially when used in conjunction with multiple biomarkers. See **Table 6** and **Figure 6**. See **Table 9** and **Figure 7**.

**Table 9:** ROC curve analysis of *CLEC4E*'s diagnostic value in SLE at different disease stages.

Factors	AUC	Sensitivity (%)	Specificity (%)	Optimal cutoff value	95% CI
C3	0.8172	64.08	85.78	0.4986	0.7715-0.8630
C4	0.9182	95.77	78.44	0.7421	0.8889-0.9475
ANA	0.5916	78.87	39.45	0.1832	0.5325-0.6507
Anti-dsDNA Antibody	0.7688	84.51	63.3	0.4781	0.7211-0.8166
*CLEC4E*	0.6360	44.37	79.36	0.2373	0.5765-0.6956
Combined Diagnosis	0.9407	95.07	82.11	0.7718	0.9174-0.9641

### 3.10 Relationship between *CLEC4E* expression and prognosis in SLE patients

**Figure 8:**
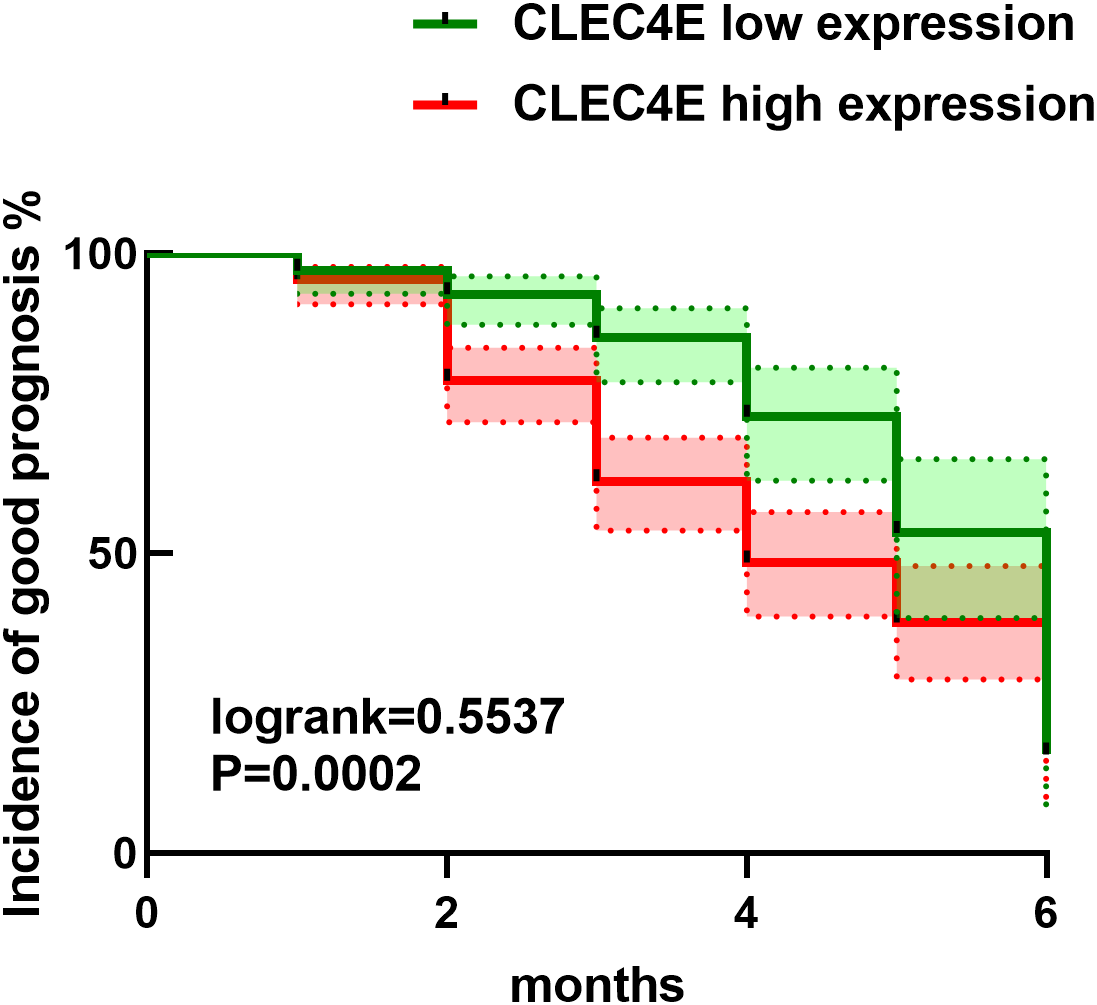
Kaplan-Meier prognosis analysis.

Kaplan-Meier survival analysis was used to assess the relationship between *CLEC4E* expression levels and prognosis in SLE patients (χ² = 13.46, *P* = 0.0002). The analysis demonstrated that patients with high *CLEC4E* expression had significantly poorer outcomes than those with low expression levels (*P* = 0.0002). These results suggest that high *CLEC4E* expression may be associated with worse clinical outcomes. See **Figure 8**.

## 4 Discussion

SLE is a highly heterogeneous and complex systemic autoimmune disease with an etiology that remains incompletely understood. Its pathological mechanisms involve various immunological abnormalities, including the production of autoantibodies, immune complex deposition, and chronic inflammatory responses (17, 18). Identifying novel biomarkers is crucial for gaining deeper insights into the pathogenesis of SLE and optimizing its diagnostic and therapeutic strategies. This study focused on *CLEC4E*, a potentially key molecule, and systematically evaluated its expression characteristics and relationship with disease-related parameters in SLE through a combination of bioinformatic analysis and clinical sample validation. Furthermore, the potential clinical applications of *CLEC4E* were explored.

Our findings demonstrated that *CLEC4E* expression was significantly elevated in SLE patients. Notably, *CLEC4E* levels were markedly higher in patients during the active phase. This finding suggests that *CLEC4E* may play a critical role in the pathophysiology of SLE. As a pattern recognition receptor, *CLEC4E* is involved in recognizing endogenous and exogenous danger signals, and its function is closely linked to the activation of the immune system (19). Within the pathological context of SLE, the elevated expression of *CLEC4E* may exacerbate chronic inflammation by enhancing the activity of neutrophils and macrophages (20). The pro-inflammatory role of *CLEC4E*, which promotes the secretion of inflammatory cytokines, could explain its strong association with disease activity indicators, such as elevated CRP levels and complement activation (21). Additionally, *CLEC4E* role in immune complex handling is significant in the context of SLE, where immune complexes are frequently deposited in tissues, contributing to organ dysfunction. *CLEC4E*’s ability to influence the handling and clearance of these immune complexes could be a crucial mechanism in mitigating inflammation and limiting tissue damage (22). The high expression of *CLEC4E* potentially reflects an abnormal inflammatory microenvironment in SLE patients, providing a theoretical basis for further exploration of SLE pathogenesis.

Further analysis revealed that *CLEC4E* expression holds promise as a clinical biomarker for both diagnosis and prognosis of SLE. The *ROC* curve analysis indicated that *CLEC4E* exhibited high sensitivity and specificity in distinguishing SLE patients from healthy individuals, with an *AUC* value of 0.7744. This finding suggests that *CLEC4E* could serve as an effective biomarker for diagnosing SLE. This is particularly significant as existing SLE diagnostic markers, while widely used, still have certain limitations in some cases. Elevated *CLEC4E* expression could complement traditional biomarkers and provide dynamic monitoring of disease progression, especially showing unique advantages in assessing disease activity. Tracking dynamic changes in *CLEC4E* expression may allow clinicians to more accurately assess fluctuations in disease status and treatment response, ultimately supporting personalized treatment strategies for patients with SLE. Additionally, this study revealed a significant association between high *CLEC4E* expression and poor prognosis in SLE patients. Kaplan-Meier survival analysis indicated that patients with elevated *CLEC4E* expression typically experienced more rapid disease progression, suggesting its potential role in predicting disease course. As a dual-function biomarker, the high expression of *CLEC4E* may not only result from enhanced inflammatory responses in the disease but also serve as a critical factor in exacerbating inflammation and tissue damage. Therefore, *CLEC4E* could be employed not only as a static diagnostic marker but also as a tool for dynamic monitoring. Regular monitoring of *CLEC4E* expression may help clinicians detect early signs of disease exacerbation, enabling timely intervention. This approach could delay disease progression, alleviate symptoms, and improve patients’ quality of life.

It is noteworthy that *CLEC4E*, as a molecule with a pivotal role in immune responses, may interact with other immunological markers, further enhancing its impact on disease progression. For example, *CLEC4E* is potentially associated with complement system activation, cytokine secretion, and the recruitment and activation of immune cells. These factors collectively drive chronic inflammation and tissue damage in SLE (23, 24). Elevated *CLEC4E* expression not only reflects excessive immune system activation but also contributes to the formation of immune pathology (25). *CLEC4E*’s influence on immune complex handling could provide a mechanistic link between immune dysregulation and tissue damage in SLE. By modulating the clearance of immune complexes, *CLEC4E* may play a key role in controlling tissue damage and inflammation (26). Therefore, CLEC4E holds promise as a potential therapeutic target. Future research could explore targeted interventions against *CLEC4E* and evaluate their potential in mitigating immune responses and reducing tissue damage in SLE patients. The potential for targeting *CLEC4E* through Mincle inhibitors is an exciting avenue for future therapeutic strategies, as inhibiting *CLEC4E* may help reduce chronic inflammation and prevent further tissue injury (20).

Despite systematically uncovering the expression characteristics and clinical significance of *CLEC4E* in SLE, this study has certain limitations. Firstly, the sample size was relatively small, and the follow-up period was limited to 6 months, which should be regarded as short-term. Larger-scale, multicenter studies with extended follow-up are required to further validate the clinical utility of *CLEC4E* and to assess the long-term prognostic value. Secondly, in the initial bioinformatics screening, multiple-testing correction (FDR) was not applied. Although this may increase the likelihood of false-positive results, our focus was to prioritize candidate genes for clinical validation, and subsequent verification in independent samples partly mitigates this concern. Thirdly, GSVA/ssGSEA or related immune deconvolution tools were not employed, which may limit our ability to comprehensively characterize the immune landscape of SLE and restrict the depth of mechanistic interpretation. In addition, although the predictive model demonstrated a high AUC, no internal validation was performed due to the limited sample size, which may raise concerns of overfitting; future studies with larger cohorts are warranted to confirm the robustness of the model. In addition, *CLEC4E* may exhibit distinct functions across different tissues or cell types, particularly in affected organs such as the kidneys and skin, where its role may differ significantly. Future studies should incorporate tissue samples or employ single-cell transcriptomics to delineate the context-specific functions of *CLEC4E*. Moreover, the mechanistic link between *CLEC4E*, complement activation, and immune complex handling remains to be fully elucidated, and clarifying these pathways may reveal how *CLEC4E* contributes to disease progression and tissue damage in SLE. Finally, while our findings suggest that *CLEC4E* and its receptor, Mincle, may serve as novel therapeutic targets, further preclinical studies are warranted to determine whether targeting this axis could attenuate inflammatory responses and ultimately improve patient outcomes.

In summary, this study is the first to systematically reveal the high expression profile of *CLEC4E* in SLE and to investigate its relationship with disease activity and prognosis. Beyond serving as a diagnostic biomarker to improve the accuracy of SLE diagnosis, *CLEC4E* may also become a crucial tool for disease monitoring and therapeutic management. Future research should explore *CLEC4E*’s functional heterogeneity across SLE subtypes using single-cell and tissue-specific analyses to support its clinical translation.

## 5 Conclusion

Increased *CLEC4E* expression correlates with higher disease activity and poorer prognosis in SLE patients, indicating its potential as a significant biomarker for disease diagnosis and monitoring.

## Data Availability

The original contributions presented in the study are included in the article/supplementary material. Further inquiries can be directed to the corresponding author.
